# Supplementary figures and images for: Multiplex in situ hybridization within a single transcript: RNAscope reveals dystrophin mRNA dynamics
Source: PLoS One. 2020 Sep 24;15(9):e0239467. doi: 10.1371/journal.pone.0239467 (PMC7514052; doi:10.1371/journal.pone.0239467)

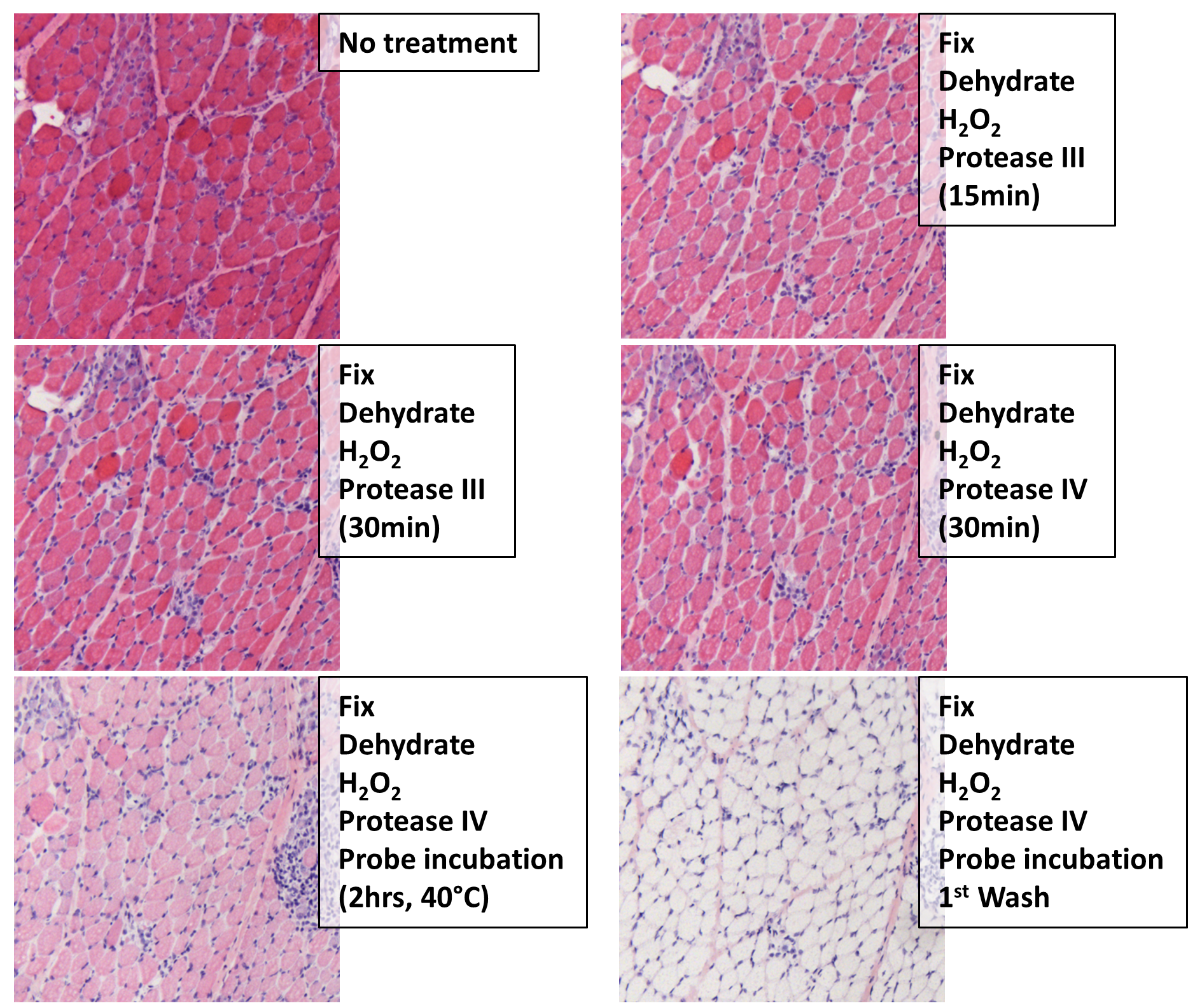

Supplement: S1 Fig — Serial slides were treated according to the protocol for fresh-frozen tissue, with a single slide taken at each indicated step and stained via Haematoxylin and Eosin. Protease treatments are well-tolerated, but muscle is visibly paler after probe incubation (lower left) and all myofibrillar material is lost entirely after a single subsequent wash. (TIF) [file pone.0239467.s001.tif]

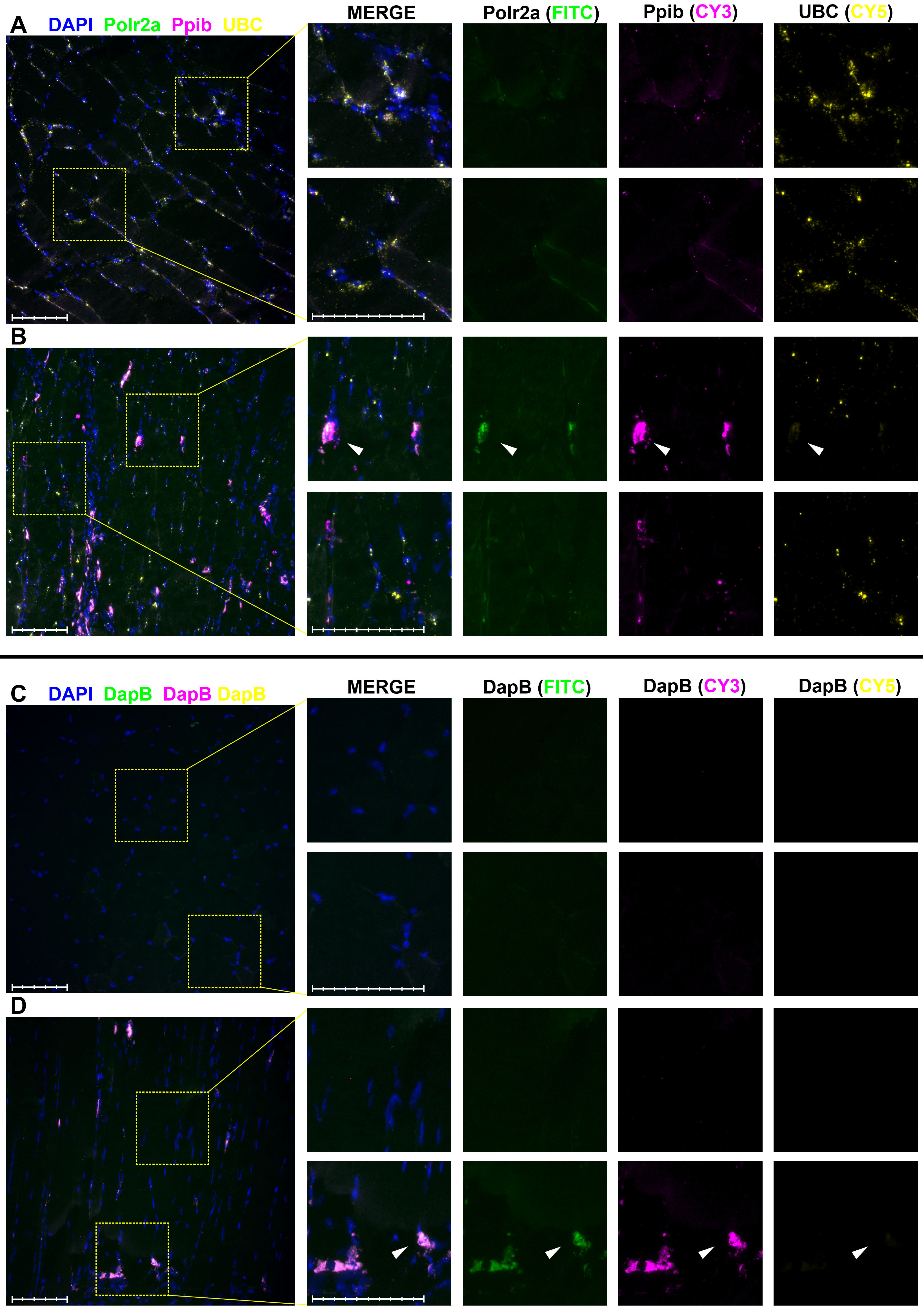

Supplement: S2 Fig — Positive control probes (Polr2a, Ppib, UBC -upper panels): Both WT (A) and mdx (B) muscle show the expected pattern: rare foci are observed for probes to Polr2a (very low abundance transcript in muscle), Ppib (low abundance in muscle) produces more foci, and staining for UBC (high abundance) is widespread. All transcripts appear to be more plentiful immediately beneath the muscle sarcolemma. Dystrophic muscle also shows patches of prominent non-specific staining (particularly in FITC and Cy3 channels) which may correspond to peroxidase-rich macrophage/neutrophil infiltrates (arrowheads). Negative control probes (bacterial DapB): WT muscle (C) shows no staining in any of the negative control probe channels. Dystrophic muscle (D) shows no probe-specific foci, but as with positive control staining (above), this tissue exhibits patches of prominent non-specific staining (particularly in FITC and Cy3 channels) which may correspond to macrophage/neutrophil infiltrates (arrowheads). Scale bars: 100μm (main panel subdivisions: 20μm; magnified panel subdivisions: 10μm). (TIF) [file pone.0239467.s002.tif]

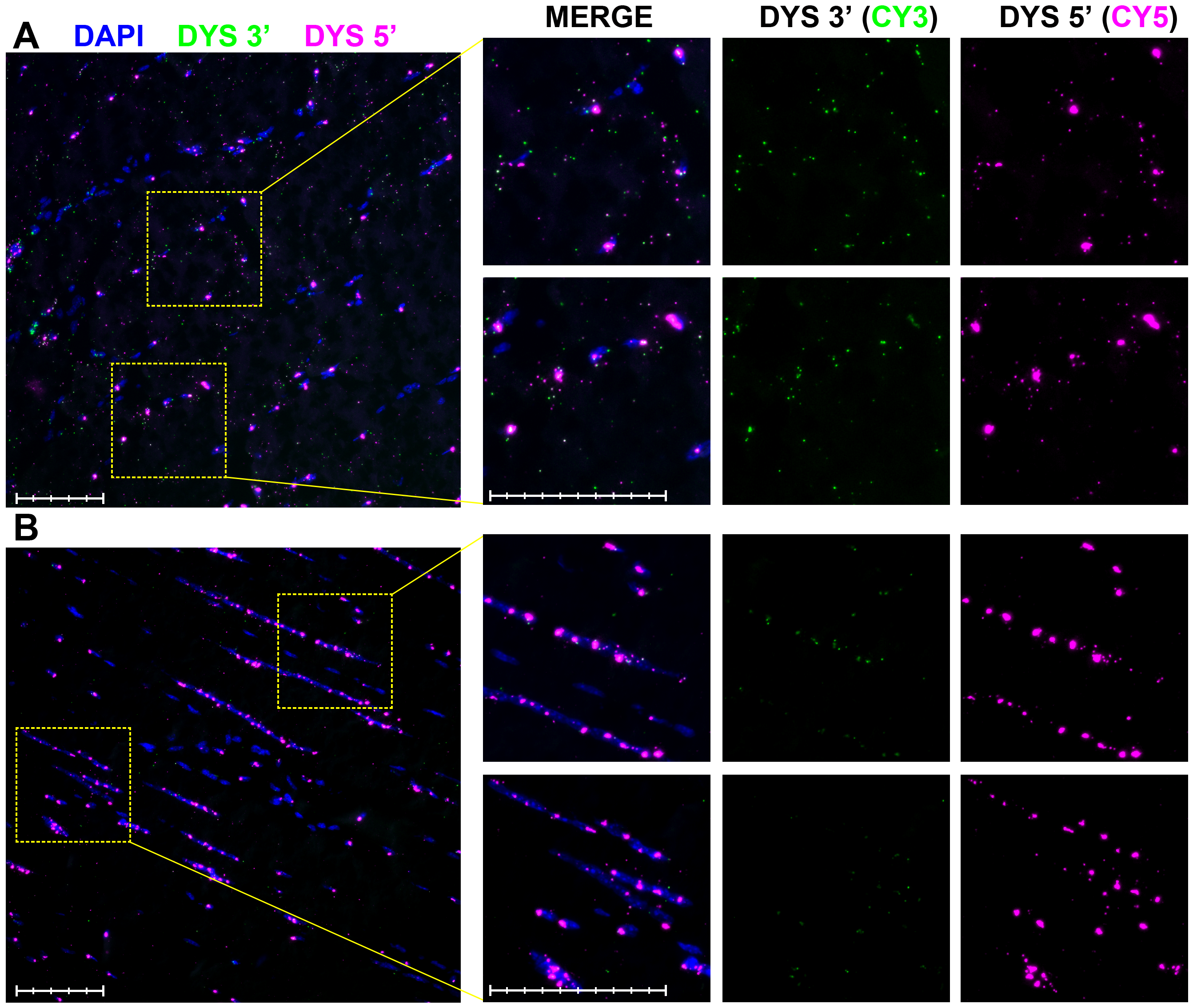

Supplement: S3 Fig — RNAscope probe labelling of 40-week old WT and mdx quadriceps muscle. Probe to dp427 5’ (Cy5: magenta) resolves both small sarcoplasmic foci and large nuclear foci, while 3’ probe (Cy3: green) shows small foci only, showing that this pattern is a property of the probes, not the fluorophores selected. Scale bars: 100μm (main panel subdivisions: 20μm; magnified panel subdivisions: 10μm). (TIF) [file pone.0239467.s003.tif]

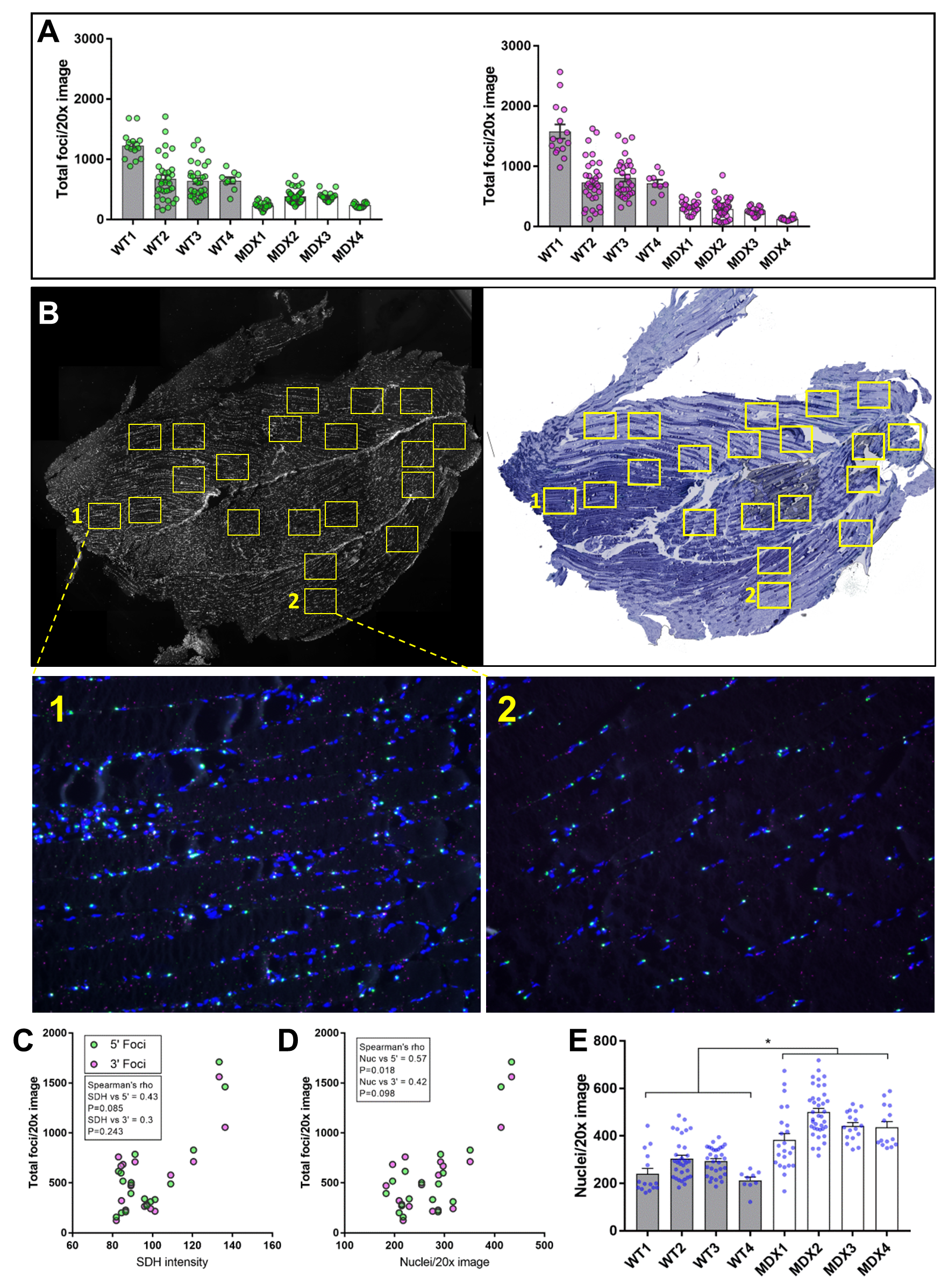

Supplement: S4 Fig — (A) total 5’ and 3’ counts per image, per individual (9–30 images per animal). (B) Alignment of RNAscope images collected with oxidative capacity: DAPI channel of RNAscope-probed WT muscle with locations of images indicated (left) and matching approximate locations in an SDH-stained serial section (right). Strongly SDH-stained regions (1) have more foci, but also more nuclei than regions of less oxidative fibres (2). SDH intensity correlates with total foci count but does not reach significance (C), while total foci count and number of nuclei per field show greater correlation (D). Images collected from mdx sections have significantly higher numbers of nuclei per field (E). Correlations: Spearman’s rho. Nuclear counts: Mann-Whitney U test using mean per image nuclear counts, per animal (N = 4 per genotype). (TIF) [file pone.0239467.s004.tif]

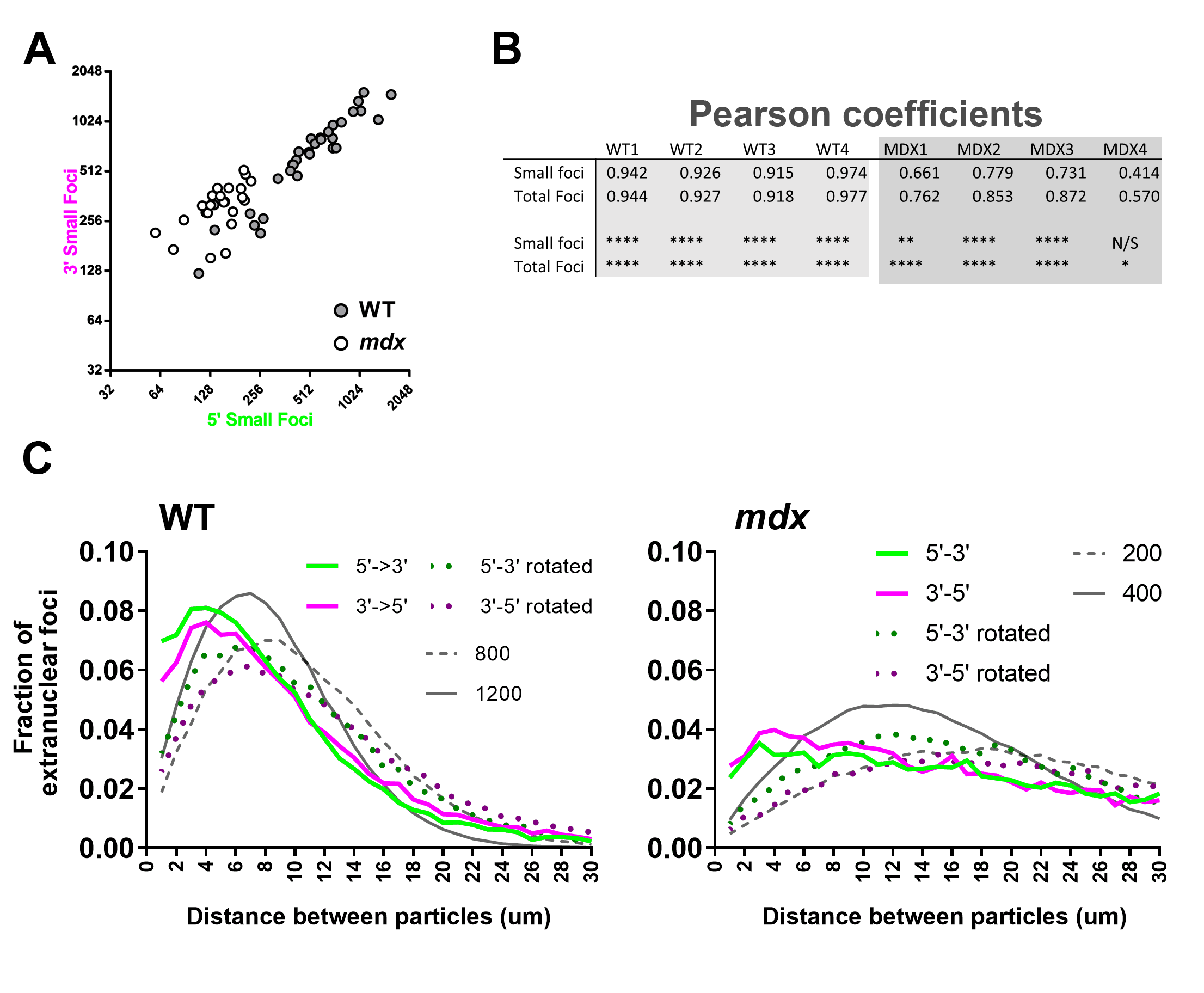

Supplement: S5 Fig — (A) Representative plot of 5’ counts vs 3’ counts for 1 healthy and 1 dystrophic individual. (B) Correlations per individual for both small foci and total foci (top rows: Pearson r values; Bottom rows: significance. * = P<0.05;** = P<0.005;*** = P<0.0005;**** = P<0.0001; N/S = not significant). (C) Distributions of nearest-neighbour distances greater than 1μm in images from WT (left) and mdx (right) muscle, compared with random distributions of the appropriate number of points (WT: 800, 1200; mdx: 200, 400) or from the same images after 3’ channel rotation. Distances <4μm are over-represented in both WT and mdx muscle. (TIF) [file pone.0239467.s005.tif]

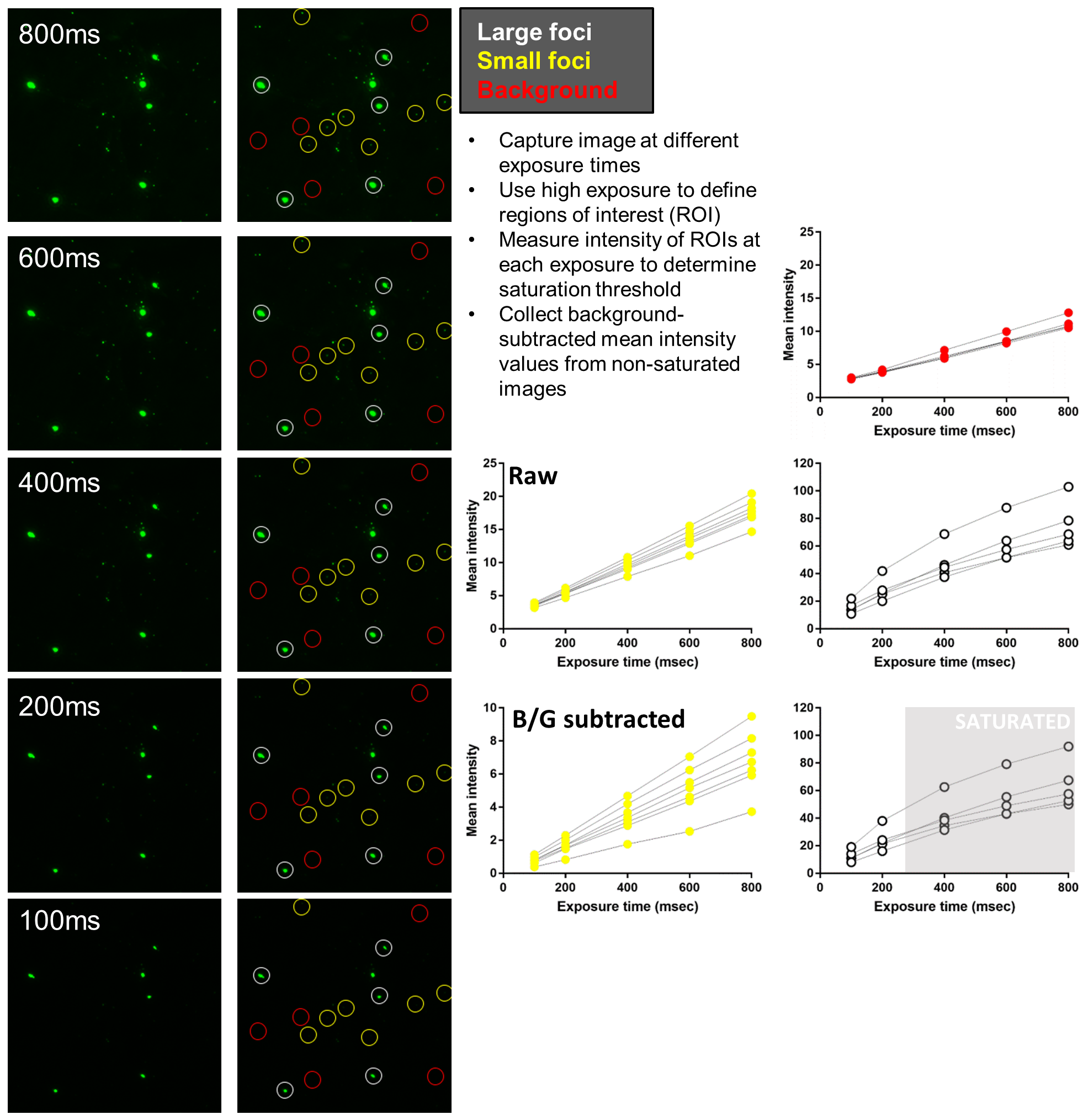

Supplement: S6 Fig — Left columns: Images of 5’ foci only were collected at 100, 200, 400, 600 and 800ms exposure times and the highest exposure images were used to define regions of interest (large foci, small foci, background). Mean fluorescence intensity in each ROI was determined for each exposure time and plotted (right). Background and small foci fluorescence values increase linearly with exposure time, whereas large foci show attenuation above 200msec, showing signal saturation. All comparisons thus used 100 and 200msec values. Images shown is cropped for clarity: all analysis used full size images with 30–50 foci of each class defined per image, using 3–5 images per individual. (TIF) [file pone.0239467.s006.tif]

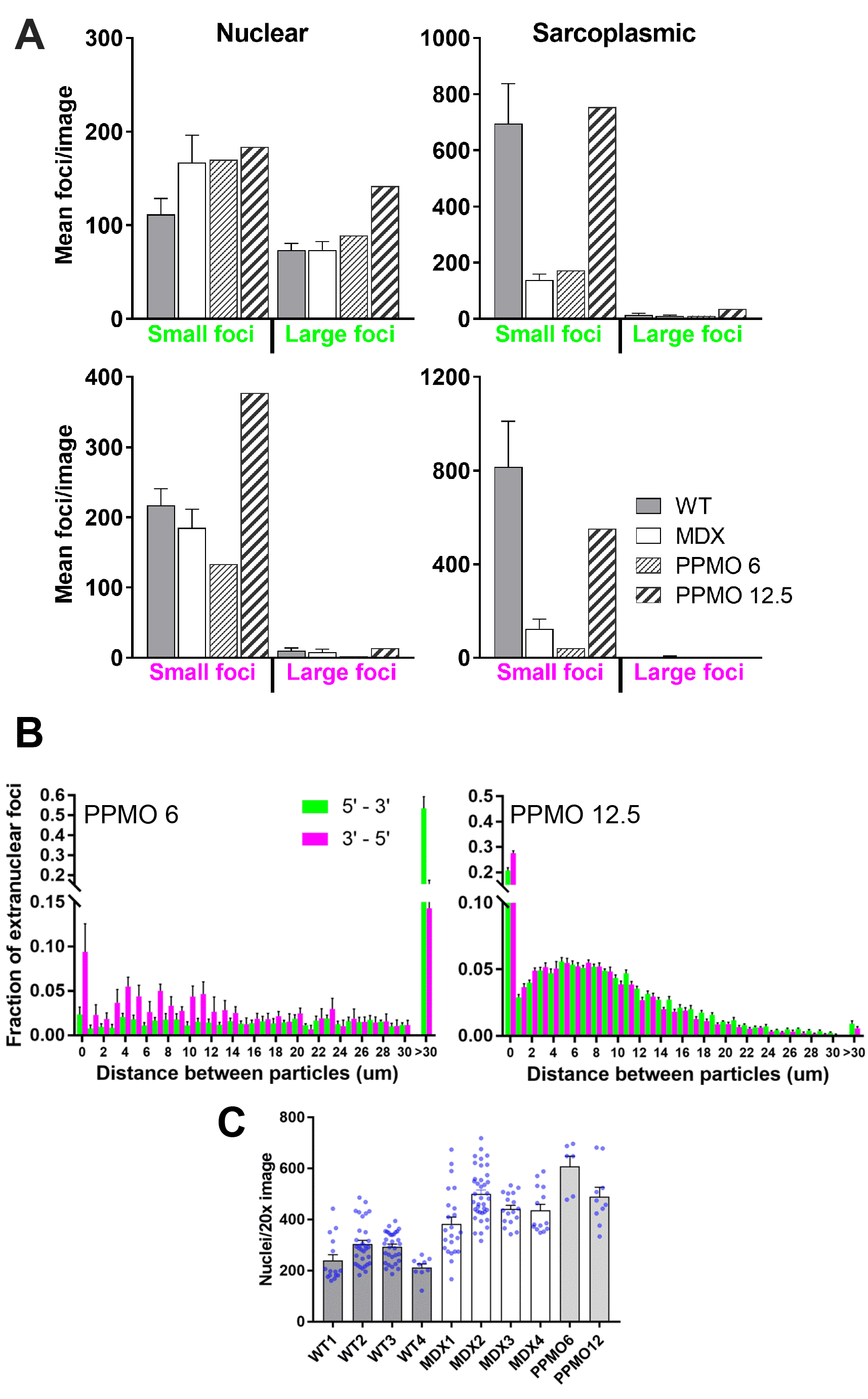

Supplement: S7 Fig — (A) Numbers and distributions of 5’ and 3’ foci in PPMO treated muscles. WT and untreated mdx values are from Fig 5. Treatment with 12.5mg.kg-1 restores small sarcoplasmic dystrophin counts to near-WT levels but also increases total nuclear counts of all foci. (B) PIP6a PMO treatment at 6mg.kg-1 does not restore probe co-localization whereas treatment at 12.5mg.kg-1 does. (C) PIP6a PPMO-treated muscles still exhibit per-image nuclear counts comparable to untreated mdx muscle. (TIF) [file pone.0239467.s007.tif]

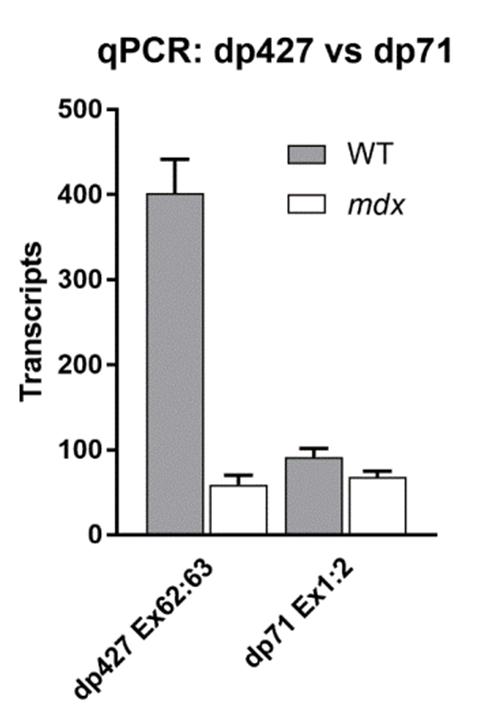

Supplement: S8 Fig — Absolute quantification of transcript numbers in WT and mdx quadriceps muscle using primers to exons 62–63 of dp427 (mature dp427) or to exons 1–2 of dystrophin isoform dp71 (dp71 unique first exon, and exon 63 of dp427). Dp71 is expressed in muscle but at low levels (50–70 transcripts per ng RNA) and is not altered in response to dystrophic pathology. Numbers of dp71 transcripts are however comparable with the greatly reduced numbers of mature dp427 transcripts in mdx muscle. (TIF) [file pone.0239467.s008.tif]
